# Associations Between Antenatal Care and Information and Pregnant Women’s Preferred Mode of Birth in Romania: A Cross-Sectional Study

**DOI:** 10.3390/healthcare14203481

**Published:** 2026-10-16

**Authors:** Mihaela Corina Radu, Laura Ioana Chivu, Letitia Draghici Goraneanu, Justin Aurelian, Raluca Elena Hanu, Sebastian Toma, Loredana Sabina Cornelia Manolescu

**Affiliations:** 1 Department of Nursing, Faculty of Midwifery and Nursing, “Carol Davila” University of Medicine and Pharmacy, 020021 Bucharest, Romania; justin.aurelian@umfcd.ro; 2 Emergency Hospital County, Str. Găgeni No. 100, 100409 Ploieşti, Romania; goraneanu.letitia@yahoo.com; 3 Department of Pathophysiology, Faculty of Midwifery and Nursing, “Carol Davila” University of Medicine and Pharmacy, 050474 Bucharest, Romania; laura.chivu@umfcd.ro; 4 “Prof. Dr. Th. Burghele” Clinical Hospital, 061344 Bucharest, Romania; 5 CMI Dr. Nicolau, 19 Dinicu Golescu Boulevard, 010865 Bucharest, Romania; raluca_hanu@yahoo.com; 6 Department of General Surgery, Faculty of Midwifery and Nursing, “Carol Davila” University of Medicine and Pharmacy, 020021 Bucharest, Romania; 7 Department of Microbiology, Parasitology and Virology, Faculty of Midwifery and Nursing, “Carol Davila” University of Medicine and Pharmacy, 020021 Bucharest, Romania; loredana.manolescu@umfcd.ro

**Keywords:** caesarean section, birth preferences, antenatal education, midwifery care, informed choice, Romania

## Abstract

**Highlights:**

**What are the main findings?**

- Among parous women, previous mode of birth was associated with birth preference; in the whole sample, preference was also associated with the information about childbirth provided by physicians. Limited antenatal information and fewer consultations were associated with greater indecision while antenatal education was associated with more frequent preferences for vaginal birth.

**What are the implications of the main findings?**

- Strengthening antenatal education and access to midwife-led counselling may support informed decision-making regarding mode of birth.
- Tailored information and timely support are particularly important for women with limited antenatal care exposure who remain undecided about childbirth.

**Abstract:**

Objectives: Romania has one of the highest caesarean rates in the European Union, yet little is known about what shapes women’s preferred mode of birth there. This study examined how socio-demographic, obstetric and antenatal care characteristics relate to preferred mode of birth, with a focus on modifiable factors such as the information received during pregnancy, attendance of antenatal classes and access to midwifery care. Methods: A cross-sectional questionnaire study of 1301 pregnant women recruited online was conducted in Romania between January and July 2023. The association between each characteristic and preferred mode of birth was tested with Pearson’s chi-square test, and effect size was quantified with Cramér’s V. *p*-Values were adjusted for multiple comparisons with the Bonferroni method, and factors independently associated with preference were identified with multinomial logistic regression (reference category: vaginal birth). Results: One-third of the women preferred caesarean birth (33.4%), just under one-third preferred vaginal birth (29.9%), and 36.7% were undecided. Among parous women, preference was associated with the mode of a previous birth (V = 0.42); in the whole sample, the largest effect size was for the information the physician gave about the birth process (V = 0.36), followed by information about breastfeeding (V = 0.24), the number of antenatal consultations (V = 0.22) and information about early skin-to-skin contact (V = 0.21). Socio-demographic factors showed weaker associations. After Bonferroni adjustment, place of residence and perceived usefulness of access to a midwife were no longer significant. In the multivariable model (n = 1301), a previous caesarean (aOR 5.06), thorough physician information about birth (aOR 2.77) and age of 30 years or over were independently associated with preferring caesarean, whereas antenatal class attendance (aOR 0.35) and thorough information about breastfeeding (aOR 0.40) were associated with lower odds of preferring caesarean. Indecision clustered among women who had received little information and had fewer consultations. Conclusions: In this predominantly urban and highly educated online sample, preferred mode of birth was associated with previous mode of birth, with potentially modifiable antenatal care and information exposure and with some socio-demographic characteristics. Given the cross-sectional design, these associations are not causal, and whether midwife-led antenatal education and counselling support informed choice should be tested in longitudinal or interventional studies.

## 1. Introduction

Caesarean birth rates have risen steadily across high-, middle- and low-income countries, and the rise is only partly explained by clinical need [1]. Globally, the procedure already accounts for more than one in five births, and projections point to nearly one in three by 2030 [1]. The World Health Organization has warned that rates above the level needed for the safety of mothers and newborns bring no additional benefit at population level and carry avoidable risks [2]. Romania sits at the high end of this trend, with one of the highest rates in the European Union, around 44% of live births, and a number of caesareans per head of population well above the European average [3]. That figure is far from the 15% to 20% range that European perinatal medicine and midwives’ associations recommend at country level [4]. The national health information system also lacks a distinct code for caesarean on maternal request, which leaves the non-clinical part of this rise without a direct measure. Against this background, how a woman comes to prefer a mode of birth is not a matter of personal taste; it is shaped by the information, care and options offered during pregnancy, and it is reflected in national rates of surgical birth.

Maternity care in Romania is strongly centred on the obstetrician and the hospital. Antenatal consultations are provided mainly by the family physician and the obstetrician-gynaecologist, within a defined minimum number of reimbursed visits, and the written information given to pregnant women through standard materials covers modes of birth only sparingly. The midwifery profession is recognised by law, and the article of Directive 2005/36/EC describing midwives’ activities, as amended by Directive 2013/55/EU, is transposed into national law through Government Emergency Ordinance 144/2008 [5,6]. The framework for practice remains thin, however. The transposing act does not define where a midwife may work and does not set out the limits of her clinical acts, and methodological norms, protocols and guidelines specific to the profession are largely absent. Midwifery education declined after 2008, so that by 2024 only two university centres still ran undergraduate admissions, with the Bucharest programme reopened in 2023. Only since 2024 has regulation required a minimum number of midwives or obstetrics-gynaecology nurses in obstetrics-gynaecology units [7]. Midwives are therefore rarely the first point of contact in pregnancy, and midwife-led continuity of care, linked in other systems with lower intervention rates [8], is scarce.

Little is known about what shapes Romanian women’s preference for mode of birth, and whether that preference is dependent on who the woman is, by age, education or place of residence, or whether it stays open to the information and care she receives during pregnancy. The distinction matters for practice. Fixed characteristics cannot be changed by a health service, but the information received, the number of consultations, attendance of structured antenatal education and contact with a midwife are modifiable. If preference follows mainly modifiable factors, then antenatal care itself becomes a means of supporting informed choice and, where appropriate, physiological birth.

This study examined the associations between a broad set of socio-demographic, obstetric and antenatal care characteristics and preferred mode of birth in a large sample of pregnant women in Romania. We looked in particular at the information received from the care provider, attendance of antenatal classes, and knowledge of and attitudes towards midwifery care, and we quantified the strength of each association to identify the most promising targets for intervention. The analysis draws on the same dataset for which the attitude instrument was psychometrically validated [9]; the predictors of the decision to opt for caesarean section and women’s perceptions of midwifery care are addressed in separate papers [10,11]. Here, we analysed preference across all three response options, caesarean, vaginal or undecided, together with its modifiable correlates. The three papers below addressed different questions, and their analyses are not repeated here. The validation study [9] tested the psychometric properties of the attitude items. The analysis of the caesarean decision [10] compared women who preferred caesarean with the whole sample, using a binary logistic model with fear of labour pain, the belief that babies born by caesarean are healthier and TORCH screening as predictors. The study on midwifery care [11] had women’s perceptions of and attitudes towards midwifery care as its outcome. In the present paper, the outcome has three categories, so that undecided women are analysed as a separate group. The exposures of interest are antenatal care and information, and all socio-demographic, obstetric and antenatal care variables are entered together in a multinomial model, with effect sizes and correction for multiple comparisons. Table 1 and the caesarean column of Table 2 repeat data already reported in [10,11] and are included only as background for the new analyses.

## 2. Materials and Methods

### 2.1. Design

This paper presents a cross-sectional, observational study based on a questionnaire self-completed by pregnant women. This is a secondary analysis of data collected in 2023 and already used in three earlier papers [9,10,11]; the differences from those papers are described in the Introduction. Reporting follows the STROBE recommendations for observational studies [12].

### 2.2. Setting and Recruitment

Participants were recruited from the general population of Romania. The questionnaire did not record the county of residence, so the geographical distribution of participants could not be determined; only the type of residence (urban or rural) was collected. Recruitment was conducted online. A notice describing the study and the measures taken to ensure anonymity was distributed through communication channels commonly used by pregnant women, including social media platforms, Facebook groups and pages dedicated to pregnant women and mothers, online parenting groups and forums, websites and social media pages of clinics and maternity hospitals, as well as groups associated with prenatal education courses. The study invitation was also disseminated during prenatal education courses and through healthcare professionals, including obstetricians, midwives, and family physicians. Interested women accessed the questionnaire through a link provided in the study invitation.

The questionnaire was administered on the Google Forms platform and remained available for seven months, between January and July 2023. Recruitment was closed in July 2023, after seven months; the closing date was not based on a sample size target.

### 2.3. Participants

Eligible women were pregnant, at least 18 years old and Romanian citizens, who agreed to take part and completed the questionnaire during pregnancy. The form could be submitted only after the mandatory items had been answered; a few items could nevertheless be left blank, so that 12 women did not state a preferred mode of birth and up to 8 women did not answer individual information items. Because Google Forms records a response only when it is submitted, questionnaires that were started but abandoned before submission were not saved; the number of women who opened the form without submitting it could therefore not be determined, and no partially completed records required handling. Of 1319 responses collected, 18 were excluded because the respondent was under 18, leaving an analytic sample of 1301 women.

Sample size. No formal a priori sample size calculation was performed before data collection. The final sample consisted of 1301 participants who met the predefined eligibility criteria and completed the questionnaire during the seven-month recruitment period. As a post hoc descriptive justification of the achieved sample size, the formula for a single proportion, n = Z^2^·*p*(1 − *p*)/d^2^, with *p* = 0.50, a 95% confidence level (Z = 1.96), and a 5% margin of error, yields a minimum sample size of 385 participants. This is not a power calculation. It shows only how many participants are needed to estimate a single proportion with a 5% margin of error, and it says nothing about the power of the chi-square tests or of the regression model. For the multinomial model, the smallest outcome group (vaginal birth, n = 389) gave about 15 events per parameter (25 parameters per contrast), above the usual minimum of 10. The achieved sample of 1301 participants substantially exceeded this conventional minimum. Given the observational and cross-sectional design, the results should nevertheless be interpreted in light of the sampling strategy and potential selection bias.

### 2.4. Questionnaire

The questionnaire covered socio-demographic characteristics (age, place of residence, education, occupation, marital status), obstetric history (parity, mode of any previous birth, current trimester, number of antenatal consultations), exposure to antenatal care and information (attendance of antenatal classes, as well as whether and how thoroughly the attending physician had discussed the birth process, early skin-to-skin contact, the first hour after birth and breastfeeding), knowledge of and attitudes towards midwifery care, and preferred mode of birth, recorded as caesarean, vaginal or undecided. A set of Likert-type items measuring attitudes towards mode of birth was developed and validated separately; its psychometric properties are reported in the instrument validation paper [9]. Age was grouped by decade (under 20, 20 to 29, 30 to 39 and 40 years or over); women aged 40 years or over were grouped together because this threshold corresponds to very advanced maternal age, which carries a distinct obstetric risk profile, and because the group was small (n = 42). The questionnaire did not collect clinical information on pregnancy risk status, complications in the current or previous pregnancies, or the clinical indication for a previous caesarean section.

### 2.5. Ethical Considerations

The study was approved by the Ethics Committee of the “Dr. Constantin Andreoiu” Emergency County Hospital, Ploiești, Romania (approval no. 41482 of 9 August 2022), and procedures followed the standards of the Declaration of Helsinki. Informed consent was obtained electronically, through the women’s choice to continue completing the questionnaire after reading the study description.

### 2.6. Statistical Analysis

Categorical variables were described as frequencies and percentages. The association between each characteristic and preferred mode of birth was tested with Pearson’s chi-square test. The strength of each association was quantified with Cramér’s V, interpreted with the usual thresholds of about 0.1, 0.3 and 0.5 for small, moderate and large effects. Where expected frequencies fell below five, results were checked with Fisher’s exact test, and small categories were merged before testing; the youngest age group was merged with the 20 to 29 group, and the analysis of previous birth mode was restricted to women with previous births. Women who reported no antenatal consultations (n = 20) were merged with the 1 to 5 group, forming a 0 to 5 category. A two-sided alpha of 0.05 was used. Women who did not answer the preferred-mode question (n = 12) were grouped with the undecided women, since neither they nor the women who answered “I do not know” had stated a preference for a particular mode of birth when they completed the questionnaire. The 12 women made up 0.9% of the sample and 2.5% of the undecided group (12/477), so their inclusion is unlikely to have changed the estimates to any important degree. Missing answers to the information items (n ≤ 8 per item) and answers indicating that the woman had not yet seen a doctor (n = 16 to 20 per item) were grouped with the “absent/not discussed” category. Because 16 bivariate comparisons were performed, *p*-values were additionally adjusted with the Bonferroni method (adjusted significance threshold 0.05/16 = 0.003). To identify factors independently associated with preferred mode of birth and to account for confounding, a multinomial logistic regression model was fitted with preferred mode of birth (caesarean, vaginal, undecided) as the dependent variable and vaginal birth as the reference category. All socio-demographic, obstetric and antenatal care variables were entered simultaneously; because mode of previous birth applies only to parous women, parity and previous mode of birth were combined into a single variable (nulliparous; previous vaginal birth only; any previous caesarean). Collinearity between the information variables was checked with variance inflation factors, and model fit was assessed with the likelihood-ratio test and Nagelkerke pseudo-R^2^. All 1301 women were included in the model. None was excluded for missing data, because missing answers had already been assigned to the categories described above. Results are reported as adjusted odds ratios (aOR) with 95% confidence intervals. The overall contribution of each variable was assessed with likelihood-ratio tests comparing the full model with the model without that variable. The multinomial model was fitted in Python (statsmodels, version 0.15.0). Analyses were carried out in JASP (version 0.18.3) and R (version 4.3.3).

## 3. Results

### 3.1. Sample Characteristics

The sample characteristics are shown in Table 1. Most women were aged between 20 and 39, lived in urban areas (71.9%) and had higher education (74.8%). Just over three-fifths were nulliparous (61.5%), and most completed the questionnaire in the third trimester (60.8%). One-third preferred caesarean birth (33.4%), just under one-third preferred vaginal birth (29.9%), and 36.7% had not yet decided (Table 1). The undecided group comprised 465 women who answered “I do not know” and 12 women who did not answer the question. Almost all participants were of Romanian ethnicity (96.8%). Most planned to give birth in a public hospital (70.4%; private hospital 26.1%), and antenatal care was provided mainly by an obstetrician-gynaecologist in the private (43.7%) or public (29.4%) system, or in both (20.8%). Among the 501 women with previous births, 199 (39.7%) had had a caesarean, 272 (54.3%) a vaginal birth and 30 (6.0%) both modes, including vaginal birth after caesarean.

**Table 1:** Characteristics of the study sample.

Characteristic	Category	n (%)
Age (years)	<20	16 (1.2)
	20–29	673 (51.7)
	30–39	570 (43.8)
	≥40	42 (3.2)
Place of residence	Rural	365 (28.1)
	Urban	936 (71.9)
Education level	Primary	28 (2.2)
	Lower secondary	48 (3.7)
	Upper secondary	252 (19.4)
	Higher	973 (74.8)
Occupation	Stable employment	987 (75.9)
	Occasional	24 (1.8)
	Not employed	290 (22.3)
Marital status	Married	1060 (81.5)
	Unmarried	224 (17.2)
	Divorced/widowed	17 (1.3)
Ethnicity	Romanian	1260 (96.8)
	Hungarian	29 (2.2)
	Roma	12 (0.9)
Planned place of birth	Public hospital	916 (70.4)
	Private hospital	340 (26.1)
	Other	45 (3.5)
Parity	No previous birth	800 (61.5)
	1–2	464 (35.7)
	3–4	33 (2.5)
	≥5	4 (0.3)
Mode of previous birth (women with previous births, n = 501)	Caesarean	199 (39.7)
	Vaginal	272 (54.3)
	Both/vaginal after caesarean	30 (6.0)
Trimester of pregnancy	First	170 (13.1)
	Second	340 (26.1)
	Third	791 (60.8)
Antenatal consultations	None	20 (1.5)
	1–5	416 (32.0)
	6–10	409 (31.4)
	11–15	298 (22.9)
	>15	158 (12.1)
Attendance of antenatal classes	Yes	476 (36.6)
	No	825 (63.4)
Physician information about the birth process	Thorough	470 (36.1)
	Brief	357 (27.4)
	Absent/not discussed	474 (36.5)
Knowledge of midwifery services	Well informed	301 (23.1)
	Partial knowledge	582 (44.7)
	Not informed	418 (32.1)
Access to a midwife seen as useful	Yes	1056 (81.2)
	No	48 (3.7)
	Unsure	197 (15.1)
Preferred mode of birth	Caesarean	435 (33.4)
	Vaginal	389 (29.9)
	Undecided/no answer *	477 (36.7)

* Includes 465 women who answered “I do not know” and 12 women who did not answer the question. Percentages for mode of previous birth are calculated among women with previous births.

### 3.2. Factors Associated with Preferred Mode of Birth

Every characteristic examined was significantly associated with preferred mode of birth in the unadjusted analysis; after Bonferroni adjustment, place of residence (adjusted *p* = 0.42) and perceived usefulness of access to a midwife (adjusted *p* = 0.06) were no longer significant. The strength of the associations varied widely (Table 2). In the whole sample, the largest effect size was for the information the attending physician gave about the birth process (V = 0.36). Among women who reported having been informed thoroughly, only 11.3% were undecided, whereas two-thirds of those who had received no information remained undecided (67.3%). Among parous women, the mode of a previous birth showed a larger effect size (V = 0.42): women with a previous caesarean rarely preferred vaginal birth (16.1%), women with a previous vaginal birth rarely preferred caesarean (12.5%), and undecided women were far more frequent after a previous vaginal birth (40.1%) than after a previous caesarean (14.1%), an association that does not show whether the preference reflects the previous birth experience itself or the clinical indication for the earlier caesarean, which was not recorded. Information about breastfeeding (V = 0.24), the number of antenatal consultations (V = 0.22) and information about early skin-to-skin contact and the first hour after birth (V = 0.21) formed the next level. A consistent pattern was observed in all of these: more contact with the care system, and more information received from it, were consistent with much lower levels of indecision. Among women with zero to five consultations, 55.5% were undecided, compared with 20.9% of those with more than fifteen.

The direction of the association differed, however, by type of contact. More intensive medical contact was associated with a more frequent preference for caesarean. Women with more consultations preferred caesarean (47.7% at 11 to 15 consultations, against 18.8% at zero to five), and those who received thorough physician information about the birth process had a stronger preference for caesarean (51.9%). Educational and midwife-oriented exposure was associated with the opposite pattern. Women who had attended antenatal classes preferred vaginal birth (38.0% against 25.2%) and caesarean less (24.2% against 38.8%), and women who knew what midwives offer, or who saw access to a midwife as useful, leaned towards vaginal birth and were less often undecided.

Socio-demographic characteristics were significant but weak. Age (V = 0.14), education (V = 0.13), occupation (V = 0.12) and place of residence (V = 0.08) were only modestly associated with preference. Older women preferred caesarean more often, and indecision clustered among younger, unmarried, less-educated and nulliparous women, and among those in the first and second trimester. The large, undecided group was not spread evenly across the sample; it was densest exactly where antenatal information and contact were lowest (Table 2).

**Table 2:** Associations between socio-demographic, obstetric, and antenatal care characteristics and preferred mode of birth among pregnant women.

Category	Caesarean n (%)	Vaginal n (%)	Undecided n (%)
Age (χ^2^(4) = 51.6, *p* < 0.001, V = 0.14)			
20–29	170 (24.7)	237 (34.4)	282 (40.9)
30–39	248 (43.5)	139 (24.4)	183 (32.1)
≥40	17 (40.5)	13 (31.0)	12 (28.6)
Place of residence (χ^2^(2) = 7.3, *p* = 0.026, V = 0.08)			
Rural	130 (35.6)	122 (33.4)	113 (31.0)
Urban	305 (32.6)	267 (28.5)	364 (38.9)
Education level (χ^2^(4) = 42.0, *p* < 0.001, V = 0.13)			
Primary/lower secondary	16 (21.1)	12 (15.8)	48 (63.2)
Upper secondary	79 (31.3)	104 (41.3)	69 (27.4)
Higher	340 (34.9)	273 (28.1)	360 (37.0)
Occupation (χ^2^(2) = 19.7, *p* < 0.001, V = 0.12)			
Stable employment	362 (36.7)	277 (28.1)	348 (35.3)
Occasional/not employed	73 (23.2)	112 (35.7)	129 (41.1)
Marital status (χ^2^(2) = 36.5, *p* < 0.001, V = 0.17)			
Married	379 (35.8)	333 (31.4)	348 (32.8)
Unmarried/divorced/widowed	56 (23.2)	56 (23.2)	129 (53.5)
Parity (χ^2^(2) = 26.0, *p* < 0.001, V = 0.14)			
One or more previous births	195 (38.9)	165 (32.9)	141 (28.1)
No previous birth	240 (30.0)	224 (28.0)	336 (42.0)
Mode of previous birth, women with previous births (χ^2^(4) = 174.9, *p* < 0.001, V = 0.42)			
Previous caesarean	139 (69.8)	32 (16.1)	28 (14.1)
Previous vaginal	34 (12.5)	129 (47.4)	109 (40.1)
Both	22 (73.3)	4 (13.3)	4 (13.3)
Trimester of pregnancy (χ^2^(4) = 88.9, *p* < 0.001, V = 0.18)			
First	45 (26.5)	32 (18.8)	93 (54.7)
Second	82 (24.1)	85 (25.0)	173 (50.9)
Third	308 (38.9)	272 (34.4)	211 (26.7)
Antenatal consultations (χ^2^(6) = 128.2, *p* < 0.001, V = 0.22)			
0–5	82 (18.8)	112 (25.7)	242 (55.5)
6–10	146 (35.7)	125 (30.6)	138 (33.7)
11–15	142 (47.7)	92 (30.9)	64 (21.5)
>15	65 (41.1)	60 (38.0)	33 (20.9)
Attendance of antenatal classes (χ^2^(2) = 36.2, *p* < 0.001, V = 0.17)			
Yes	115 (24.2)	181 (38.0)	180 (37.8)
No	320 (38.8)	208 (25.2)	297 (36.0)
Physician information about the birth process (χ^2^(4) = 342.2, *p* < 0.001, V = 0.36)			
Thorough	244 (51.9)	173 (36.8)	53 (11.3)
Brief	121 (33.9)	131 (36.7)	105 (29.4)
Absent/not discussed	70 (14.8)	85 (17.9)	319 (67.3)
Information about early skin-to-skin contact (χ^2^(4) = 115.7, *p* < 0.001, V = 0.21)			
Thorough	119 (41.2)	133 (46.0)	37 (12.8)
Brief	57 (39.0)	49 (33.6)	40 (27.4)
Absent	259 (29.9)	207 (23.9)	400 (46.2)
Information about the first hour after birth (χ^2^(4) = 119.5, *p* < 0.001, V = 0.21)			
Thorough	70 (36.6)	109 (57.1)	12 (6.3)
Brief	44 (41.9)	29 (27.6)	32 (30.5)
Absent	321 (31.9)	251 (25.0)	433 (43.1)
Information about breastfeeding (χ^2^(4) = 152.4, *p* < 0.001, V = 0.24)			
Thorough	90 (40.2)	118 (52.7)	16 (7.1)
Brief	61 (35.9)	69 (40.6)	40 (23.5)
Absent	284 (31.3)	202 (22.3)	421 (46.4)
Knowledge of midwifery services (χ^2^(2) = 22.5, *p* < 0.001, V = 0.13)			
Informed	294 (33.3)	297 (33.6)	292 (33.1)
Not informed	141 (33.7)	92 (22.0)	185 (44.3)
Access to a midwife seen as useful (χ^2^(2) = 11.3, *p* = 0.003, V = 0.09)			
Yes	347 (32.9)	337 (31.9)	372 (35.2)
No/unsure	88 (35.9)	52 (21.2)	105 (42.9)

Percentages are calculated by row, within each category. χ^2^, chi-square statistic; V, Cramér’s V. The youngest age group (under 20 years) was merged with the 20 to 29 group before testing. The analysis of previous birth mode is restricted to women with previous births. Women reporting no antenatal consultations (n = 20) are included in the 0–5 category. Bonferroni-adjusted significance threshold: *p* < 0.003 (16 comparisons).

### 3.3. Multivariable Analysis

In the multinomial logistic regression model (Table 3), which included all 1301 women and all variables simultaneously, previous mode of birth remained independently associated with preference. Compared with nulliparous women, women with any previous caesarean had about five times higher odds of preferring caesarean rather than vaginal birth (aOR 5.06, 95% CI 3.05–8.39), whereas women with only previous vaginal births had lower odds of preferring caesarean (aOR 0.23, 95% CI 0.14–0.40) and of being undecided (aOR 0.52, 95% CI 0.33–0.82). Thorough information from the physician about the birth process was independently associated with a preference for caesarean (aOR 2.77, 95% CI 1.55–4.97) and with much lower odds of indecision (brief: aOR 0.31, 95% CI 0.19–0.48; thorough: aOR 0.19, 95% CI 0.10–0.35). In contrast, attendance of antenatal classes (aOR 0.35, 95% CI 0.23–0.53), thorough information about breastfeeding (aOR 0.40, 95% CI 0.21–0.79) and thorough information about the first hour after birth (aOR 0.42, 95% CI 0.19–0.95) were associated with lower odds of preferring caesarean; the two information variables were also strongly associated with lower odds of indecision (breastfeeding: aOR 0.15, 95% CI 0.06–0.35; first hour: aOR 0.10, 95% CI 0.03–0.27). Women with 11 to 15 antenatal consultations had higher odds of preferring caesarean (aOR 1.82, 95% CI 1.05–3.15), whereas the other consultation categories were not independently associated with preference. Pregnancy risk status was not recorded, and women with high-risk or complicated pregnancies usually have more consultations, so this result may partly reflect clinical need (see Section Strengths and Limitations). Among socio-demographic factors, age of 30 to 39 years (aOR 2.73, 95% CI 1.90–3.92) and 40 years or over (aOR 4.19, 95% CI 1.64–10.71) remained associated with a preference for caesarean, while urban residence (aOR 0.62, 95% CI 0.42–0.92), upper secondary education (aOR 0.35, 95% CI 0.21–0.57) and occasional or no employment (aOR 0.52, 95% CI 0.33–0.81) were associated with lower odds of preferring caesarean. Women in the first trimester had higher odds of preferring caesarean (aOR 2.51, 95% CI 1.27–4.97). Indecision was independently associated with being unmarried, divorced or widowed (aOR 4.17, 95% CI 2.50–6.96), primary or lower secondary education (aOR 3.32, 95% CI 1.35–8.17), and the first (aOR 4.03, 95% CI 2.11–7.66) and second trimester (aOR 2.13, 95% CI 1.36–3.33), and was less frequent among women who saw access to a midwife as useful (aOR 0.47, 95% CI 0.29–0.76). Knowledge of midwifery services was not independently associated with preference (likelihood-ratio test, *p* = 0.81). After adjustment, thorough information about skin-to-skin contact was associated with higher odds of indecision (aOR 4.22, 95% CI 1.64–10.87), contrary to the unadjusted pattern; because this variable was correlated with the other information variables, this estimate should be interpreted with caution. A likely explanation is the overlap between the information items. Skin-to-skin contact, the first hour after birth and breastfeeding are related topics that are often discussed together, so once the three items are adjusted for each other, the estimate for skin-to-skin contact rests on the few women whose information on this topic differed from their information on the other two. Estimates of this kind can change sign even when variance inflation factors are low (a suppression effect), and the wide confidence interval points the same way. We therefore do not read this result as showing that information about skin-to-skin contact makes women more undecided. The model fitted the data significantly better than the intercept-only model (likelihood-ratio χ^2^(50) = 860.3, *p* < 0.001; Nagelkerke R^2^ = 0.54), and no relevant collinearity was found (all variance inflation factors < 4).

## 4. Discussion

In this large sample of pregnant women in Romania, preferred mode of birth was associated both with women’s obstetric history and with what happened during antenatal care, more than with most socio-demographic characteristics. The information received from the physician about the birth process showed the largest bivariate effect size for preference in the whole sample, ahead of any socio-demographic characteristic. Because information and antenatal contact can be changed, the result points to antenatal care as the place where informed choice can be supported. This fits the findings that link the way professionals communicate and the content of discussions during pregnancy to women’s preferences [13], and a counselling intervention based on shared decision-making has been shown to change the preferences women express [14].

The most useful distinction the data offer is between how much antenatal contact a woman has and what kind. More contact, from any source, was associated with less indecision; women who interact often with services have more chances to form and state a preference. The direction of that preference is split by the type of contact. Frequent medical visits and detailed physician information was associated with a stronger leaning towards caesarean, and structured antenatal education and midwife orientation with a stronger leaning towards vaginal birth. Part of this link may also be clinical: women with high-risk or complicated pregnancies are seen more often and are more likely to need a caesarean, but these data were not collected (Section Strengths and Limitations). After adjustment, antenatal class attendance remained independently associated with lower odds of preferring caesarean, whereas knowledge of midwifery services was no longer independently associated with preference. One plausible reading is that, in a system with many interventions, routine obstetric contact comes to present surgical birth as ordinary, while antenatal education and midwifery care present physiological birth as a supported and realistic option. Recent syntheses show that the decision for caesarean is shaped strongly by clinicians’ beliefs and by the features of the system they work in [15], and that few women prefer caesarean in the absence of medical reasons [16]. Antenatal preference tends to form where the system is medically dominated, and the caesarean rate is high [17]. This interpretation remains an assumption and should be tested directly.

The close link between a previous caesarean and a preference for a repeat caesarean deserves particular attention in a setting with a rate near 44%. This association should not, however, be read as evidence that a preference for repeat caesarean results from inadequate counselling. The indication for the previous caesarean, whether recurrent (for example, a contracted pelvis) or non-recurrent (for example, fetal distress or breech presentation), the number of previous caesareans, the interval between births and any contraindication to a trial of labour all legitimately influence the mode of the next birth, and for many women a planned repeat caesarean is the clinically appropriate or an informed choice. These data were not collected in our study, so we could not distinguish women for whom vaginal birth after caesarean would have been a safe option from those for whom it was not. For women who are eligible for a trial of labour, the absence of an active discussion about vaginal birth after caesarean may make each surgical birth more likely to be followed by another, and preference takes shape early in pregnancy. Women with a previous caesarean build their choice from experience and from how they understand why the first operation was needed, and professional support weighs visibly here [13,18]. Counselling based on shared decision-making reduces decisional conflict and increases the proportion of women who choose and achieve a vaginal birth after caesarean [19]. The midwife is well placed to open this discussion, together with the obstetrician who assesses eligibility for a trial of labour, during pregnancy.

The size of the undecided group, more than one in three women, is itself a result. Indecision was not spread at random. It gathered among women who had received little information, who had been seen less often, and who were younger, unmarried, less-educated or early in pregnancy. These are the women whose preferences are still open and whom the care system reaches least. For them, midwife-led antenatal counselling could matter most, because it supports a genuinely informed decision and reaches women otherwise at the margins of care. Uncertainty around mode of birth clears more easily when a woman receives good information in good time, and unhurried support [14,19].

The modifiable factors identified here, namely information and structured antenatal education, act within a system where the midwife’s autonomous role is thinly defined in law and the workforce is small. The transposition of the midwifery directive is not matched by norms setting out places of practice and the limits of competence, and training was in decline for a long time, which for now narrows the reach of midwife-led counselling. A few recent measures are beginning to open this space: the 2024 requirement to have midwives or obstetrics-gynaecology nurses in the relevant units [7], alongside the reopening of the university midwifery programme. Evidence from other systems is consistent: midwife-led models and continuity of care are associated with more spontaneous vaginal births and lower caesarean rates [8,20], and antenatal education supports the same direction [21]. Set against the 15% to 20% range recommended at country level [4], the national rate of about 44% shows how much room there is to act on the factors the service can change.

Our questionnaire did not ask women why they preferred a particular mode of birth, so the reasons behind each choice could not be established directly. The attitude items of the questionnaire, summarised descriptively, which come from the instrument validated in [9] and partly overlap with the predictors used in [10], nevertheless give some indirect insight. Most women (70.1%) agreed that fear of labour pain influences the choice of mode of birth, 57.6% that caesarean on request should be available to all pregnant women, and 38.7% that it is more accessible in the private sector. At the same time, 70.5% agreed that recovery is easier and faster after vaginal birth, 65.7% that birth should not be interfered with unless medically necessary, 57.8% that skin-to-skin contact is easier after vaginal birth and 52.0% that vaginal birth increases a woman’s self-confidence. Almost all women (94.4%) agreed that choosing the mode of birth is the woman’s right but must be an informed and medically approved choice, and 81.8% that better information about caesarean section would lead to more informed choices. In addition, 69.1% of women considered it very important to be actively involved in decisions about their birth and 17.6% somewhat important, whereas 13.3% preferred to leave these decisions to health professionals. These attitudes were not analysed in relation to stated preference and should be read alongside earlier studies. A preference for caesarean has been linked mainly to fear of childbirth and of labour pain, concern for the safety of the baby, a previous caesarean or a negative previous birth experience, and the attitudes and recommendations of the attending obstetrician [15,16,22,23]. A preference for vaginal birth is usually explained by the wish for a natural birth, faster recovery, earlier contact with the newborn and easier initiation of breastfeeding, and the avoidance of surgery and its risks [16,17]. Several of these explanations are consistent with our findings: women who had received thorough information about breastfeeding, skin-to-skin contact and the first hour after birth more often preferred vaginal birth in the unadjusted analysis (after adjustment, the estimate for skin-to-skin contact changed direction; see Section 3.3), and women with a previous caesarean more often preferred a repeat caesarean, in line with the higher preference for caesarean reported after a previous caesarean worldwide [22]. Indecision, in turn, was most frequent early in pregnancy and among women with little information, which suggests that for many women, preference had simply not yet formed. Future studies should measure women’s stated reasons, fear of childbirth and the clinical context directly.

### Strengths and Limitations

The main strengths are the large sample and the unusually broad set of antenatal care and information variables examined alongside socio-demographic factors, together with the use of a separately validated attitude instrument. Several limitations temper the conclusions. The design is cross-sectional, so associations cannot be read as causal, and reverse pathways are possible; a woman who already favours caesarean may seek more medical contact, rather than that contact shaping her preference. The measured outcome is a preference stated during pregnancy, not the mode of birth that actually took place. The data are self-reported and open to recall and social desirability effects. Because many characteristics were tested, some associations may reflect multiple comparisons, although the pattern of effect sizes, not significance alone, guides interpretation, and the largest effects are unlikely to be chance. To address this, *p*-values were additionally adjusted with the Bonferroni method and a multivariable model was fitted. The directions observed for antenatal education and midwife orientation are associative and do not re-test the companion analyses of the caesarean decision or of perceptions of midwifery care. The sample was well-educated and largely urban, which limits how far the results extend to rural and less-educated women, the very groups in which indecision was highest. Recruitment was online and voluntary, so the sample is self-selected and over-represents urban, highly educated and employed women with internet access; the findings should therefore be interpreted with caution and cannot be generalised to all pregnant women in Romania. Clinical information such as pregnancy risk status, complications in the current or previous pregnancies and the indication for a previous caesarean was not collected, although these factors can influence both the number of antenatal consultations and the preferred mode of birth and may confound some of the associations observed. This matters for the regression results. Women with high-risk or complicated pregnancies tend to have more consultations and are more likely to need or expect a caesarean, so the higher odds of preferring caesarean at 11 to 15 consultations may reflect confounding by indication. In the same way, the link between a previous caesarean and a preference for a repeat caesarean may partly reflect recurrent indications or contraindications to a trial of labour. As such, neither association should be taken as an effect of antenatal contact. The information variables were also related to each other; variance inflation factors were acceptable, but adjusting them for one another produced at least one implausible estimate (skin-to-skin contact), so the adjusted results for single information items need cautious interpretation. Finally, the 12 women who did not answer the preference question were counted as undecided, although not answering does not always mean indecision. Women were not asked why they preferred a particular mode of birth: their attitudes towards each mode of birth were recorded but were summarised only descriptively. The sector in which antenatal care was provided (private for 43.7% of women) and the planned place of birth were also recorded but not included in the present analysis, although both may be associated with a preference for caesarean. County of residence was not recorded, so we could not assess whether all regions of Romania were represented in the sample.

## 5. Conclusions

Among pregnant women in Romania who took part in this online survey, preferred mode of birth was associated with previous mode of birth and with potentially modifiable antenatal care and information exposures; in the multivariable model, previous caesarean, thorough physician information, antenatal class attendance and information about breastfeeding and the first hour after birth remained independently associated with preference, alongside age and marital status. More frequent medical contact and thorough physician information were associated with a more frequent preference for caesarean, whereas attendance of antenatal classes was associated with a lower preference for caesarean, also after adjustment. More than one-third of women remained undecided, mostly among those with the least antenatal information and contact. Given the cross-sectional design and the predominantly urban and highly educated sample, these associations cannot be interpreted as causal. Longitudinal and interventional studies are needed to establish whether wider access to antenatal education and midwife-led counselling supports informed decision-making about mode of birth.

## Figures and Tables

**Table 3:** Multinomial logistic regression of factors associated with preferred mode of birth (reference category: vaginal birth). n = 1301.

Variable (Reference Category)	Caesarean vs. Vaginal, aOR (95% CI)	Undecided vs. Vaginal, aOR (95% CI)
Age (ref. under 30 years)		
30–39	2.73 (1.90–3.92) ***	1.37 (0.94–2.00)
≥40	4.19 (1.64–10.71) **	0.83 (0.32–2.16)
Place of residence (ref. rural)		
Urban	0.62 (0.42–0.92) *	1.16 (0.75–1.81)
Education (ref. higher)		
Primary/lower secondary	1.56 (0.61–3.96)	3.32 (1.35–8.17) **
Upper secondary	0.35 (0.21–0.57) ***	0.68 (0.42–1.12)
Occupation (ref. stable employment)		
Occasional/not employed	0.52 (0.33–0.81) **	0.71 (0.45–1.12)
Marital status (ref. married)		
Unmarried/divorced/widowed	1.07 (0.64–1.79)	4.17 (2.50–6.96) ***
Parity and previous birth (ref. nulliparous)		
Previous vaginal birth only	0.23 (0.14–0.40) ***	0.52 (0.33–0.82) **
Any previous caesarean	5.06 (3.05–8.39) ***	0.87 (0.45–1.68)
Trimester (ref. third)		
First	2.51 (1.27–4.97) **	4.03 (2.11–7.66) ***
Second	0.94 (0.59–1.52)	2.13 (1.36–3.33) ***
Antenatal consultations (ref. 0–5)		
6–10	1.45 (0.87–2.44)	0.91 (0.57–1.46)
11–15	1.82 (1.05–3.15) *	0.74 (0.44–1.26)
>15	1.83 (0.96–3.48)	0.57 (0.29–1.10)
Antenatal classes (ref. no)		
Yes	0.35 (0.23–0.53) ***	1.14 (0.75–1.73)
Physician information about the birth process (ref. absent)		
Brief	0.99 (0.60–1.64)	0.31 (0.19–0.48) ***
Thorough	2.77 (1.55–4.97) ***	0.19 (0.10–0.35) ***
Information about skin-to-skin contact (ref. absent)		
Brief	0.84 (0.46–1.53)	1.08 (0.57–2.04)
Thorough	1.00 (0.44–2.27)	4.22 (1.64–10.87) **
Information about the first hour after birth (ref. absent)		
Brief	1.38 (0.67–2.83)	1.29 (0.61–2.73)
Thorough	0.42 (0.19–0.95) *	0.10 (0.03–0.27) ***
Information about breastfeeding (ref. absent)		
Brief	0.54 (0.32–0.93) *	0.41 (0.24–0.71) **
Thorough	0.40 (0.21–0.79) **	0.15 (0.06–0.35) ***
Knowledge of midwifery services (ref. not informed)		
Informed	1.12 (0.73–1.71)	1.14 (0.75–1.74)
Access to a midwife seen as useful (ref. no/unsure)		
Yes	0.63 (0.40–1.01)	0.47 (0.29–0.76) **

aOR, adjusted odds ratio; CI, confidence interval. All variables were entered simultaneously, n = 1301; * *p* < 0.05; ** *p* < 0.01; *** *p* < 0.001. Likelihood-ratio χ^2^(50) = 860.3, *p* < 0.001; Nagelkerke R^2^ = 0.54; maximum variance inflation factor 3.97.

## Data Availability

The data presented in this study are available on request from the corresponding author. The data are not publicly available because they contain information that could compromise the privacy of the participants.
